# Exercise Testing in Patients with Pulmonary Hypertension

**DOI:** 10.3390/jcm13030795

**Published:** 2024-01-30

**Authors:** Anika Vaidy, Cyrus A. Vahdatpour, Jeremy Mazurek

**Affiliations:** 1 Division of Cardiology, School of Medicine, University of Pennsylvania, Philadelphia, PA 19104, USA; anika.vaidy@pennmedicine.upenn.edu; 2 Division of Pulmonary Medicine, School of Medicine, University of Pennsylvania, Philadelphia, PA 19104, USA; cyrus.vahdatpour@pennmedicine.upenn.edu

**Keywords:** pulmonary hypertension, cardiopulmonary exercise testing, hemodynamics, heart failure with preserved ejection fraction, exercise-induced pulmonary hypertension

## Abstract

Pulmonary hypertension (PH), defined by a mean pulmonary artery pressure of >20 mm Hg, often presents with non-specific symptoms such as dyspnea and exercise intolerance, making it difficult to diagnose early before the onset of right heart dysfunction. Therefore, exercise testing can be of great utility for clinicians who are evaluating patients with an unclear etiology of exercise intolerance by helping identify the underlying mechanisms of their disease. The presence of PH is associated with adverse clinical outcomes, with distinct differences and patterns in the cardiovascular and ventilatory responses to exercise across various PH phenotypes. We discuss the role of exercise-invasive hemodynamic testing, cardiopulmonary exercise testing, and exercise stress echocardiography modalities across the spectrum of PH.

## 1. Introduction

Pulmonary hypertension (PH), defined by a mean pulmonary artery pressure (mPAP) of >20 mm Hg, often presents with non-specific symptoms such as dyspnea and exercise intolerance, making it difficult to diagnose early before the onset of right heart dysfunction. Therefore, exercise testing can be of great utility for clinicians who are evaluating patients with an unclear etiology of exercise intolerance by helping identify the underlying mechanisms of their disease.

PH can be hemodynamically categorized into three distinct entities. Precapillary PH consists of a pulmonary capillary wedge pressure (PCWP) ≤ 15 mm Hg and pulmonary vascular resistance (PVR) > 2 wood units (WU), and isolated postcapillary pulmonary hypertension (Ipc-PH) has a PCWP > 15 mm Hg with a PVR ≤ 2 WU. Lastly, combined postcapillary and precapillary pulmonary hypertension (Cpc-PH) is defined by a PCWP > 15 mm Hg and a PVR > 2 WU [1].

The presence of PH is associated with adverse clinical outcomes, with distinct differences and patterns in the cardiovascular and ventilatory responses to exercise across these phenotypes. This highlights the role of exercise testing in certain patients with left heart dysfunction in identifying the presence and type of PH. Below, we discuss the role of exercise-invasive hemodynamic testing, cardiopulmonary exercise testing (CPET), and exercise stress echocardiography modalities across the spectrum of PH.

## 2. Exercise Invasive Hemodynamics in the Assessment of PH

### 2.1. Precapillary PH

Pulmonary arterial remodeling and vascular obstruction lead to increases in PVR and mPAP. In patients with longstanding pulmonary vascular disease, the elevated PVR results in increased RV wall stress, leading to cardiac myocyte hypertrophy, increased wall thickness, and augmented contractility to maintain stroke volume. With disease progression, right heart dilatation and dysfunction occur. This is accompanied by an increasing degree of tricuspid regurgitation (TR), leftward interventricular septal displacement, and subsequential reductions in left atrial and left ventricular (LV) cavity size with eventual falling cardiac stroke volume. As the RV dilates and loses function, it “uncouples” from the pulmonary circulation, and becomes unable to match the degree of RV afterload. This eventually leads to right heart failure.

Normally during exercise, cardiac output (CO) increases during exercise to accommodate for increasing oxygen demand from the peripheral muscles. There is increased pulmonary blood flow, which is usually met with vascular distention and recruitment. This adaptation preserves low vascular resistance and allows the maintenance of reduced RV afterload [2]. In PAH patients undergoing exercise, CO fails at this accommodation due to rises in mPAP and right ventricular failure (RVF). The rise in mPAP is a consequence of pulmonary vascular remodeling, which prevents normal vascular recruitment and distention during exercise, leading to an abnormal rise in measured PVR. The rise in PVR results in impaired stroke volume augmentation, making the CO during exercise reliant on heart rate.

The addition of right heart catheterization (RHC) with exercise can reveal several characteristic abnormalities in patients with PAH. There is a spectrum of findings dependent on the stage of the disease process. In early PAH or exercise-induced pulmonary hypertension (EIPH), CO augmentation may not be affected, but PVR may fail to fall appropriately. EIPH may represent a precursor stage of PH before overt signs are evident at rest. Ho et al. investigated the clinical significance of EIPH across a broad range of patient phenotypes, all of whom suffered from chronic dyspnea on exertion [3]. During the exercise, serial mPAP and CO measurements were obtained and EIPH was defined as a delta mPAP/delta CO slope > 3 mm Hg/L/min. They found that the presence of exercise PH predicts worse cardiovascular event-free survival and was associated with worse functional capacity than those without exercise PH. This held true among even those with mild resting PH (mPAP 21–29 mm Hg). Whether the identification of EIPH should prompt early initiation of treatment and/or prevent more advanced stages of the disease remains an open question. There have been small studies in patients (all of whom had normal resting hemodynamics) with systemic sclerosis and exercise-induced PH which have shown improvements in exercise hemodynamics and functional capacity in those treated with ambrisentan [4].

As mentioned previously, an increase in flow can normally distend compliant, thin-walled pulmonary vessels. A loss of the ability to maintain appropriate distension with exercise may be an early marker of pulmonary arteriopathy [5]. More specifically, distensibility is defined as the percent increase in diameter of the smallest pulmonary arteries per mm Hg increase in pressure. In healthy individuals, the increase in diameter is 1.5–2% per mm Hg. Distensibility can be calculated by using various hemodynamic parameters that are commonly obtained during an exercise RHC [6]. Various studies have illustrated the prognostic value of distensibility, for patients with lower distensibility have worse exercise capacity, right ventricular/pulmonary artery (RV:PA) coupling, and overall survival [5,6]. It is also known to be modifiable with pulmonary vasodilatory therapy and, therefore, may be an important target for future therapies in patients with EIPH.

As the pulmonary arterial hypertension (PAH) syndrome advances, there will be an increase in the PVR which can lead to an inability of the right heart to augment stroke volume with exercise. In cases of advanced PAH, the stroke volume will not rise appropriately with exercise. Additionally, the right atrial pressure will often rise along with an increase in the right atrial pressure: pulmonary capillary wedge pressure (RAP:PCWP) ratio. In some cases, as TR worsens with RV dilation during exercise, large V waves may even be appreciated in the RAP tracing.

### 2.2. Left Heart Disease-Associated PH

Left heart failure is characterized by high left-sided cardiac filling pressures at rest or with exercise. The role of exercise provocation has emerged as extremely important in identifying the presence of abnormal left-sided filling pressures and/or pulmonary pressures in the absence of abnormal findings at rest. This is especially true in those with heart failure with preserved ejection fraction (HFpEF), where a diagnosis may be elusive on routine resting echocardiogram [7]. Patients with PH related to HFpEF, by definition, have postcapillary PH. When left-sided filling pressures are within the normal range at rest, obtaining exercise hemodynamics is essential in making the diagnosis of HFpEF and unmasking the cause for dyspnea in a given patient. In a patient with Ipc-PH, there will be an increase in PCWP > 25 mm Hg and mPAP > 30 mm Hg with exertion [8], but with an appropriate fall in PVR. RA:PCWP ratio should remain <0.5, for this is a disease that is predominantly driven by left heart stiffness.

In an elegant study by Lewis et al., investigators used the PCWP/CO slope as a way to assess impaired exercise capacity in patients with undifferentiated dyspnea and normal PCWP at rest. This allows for a correction for augmentation in PCWP for the corresponding increase in cardiac flow as measured by CO. The study found that over 40% of the individuals had an abnormal PCWP/CO slope response that was revealed with exercise. Importantly, an elevated PCWP/CO slope was significantly related to impaired exercise capacity and predicted worse heart failure-free survival [9].

In at least a quarter of patients with PH-HFpEF, chronically elevated left-sided filling pressures and backward transmission to the pulmonary circulation (among other factors including other comorbid conditions), can result in pulmonary vascular remodeling and pulmonary arteriopathy. This is reflected by an elevated PVR and described as Cpc-PH. Total pulmonary resistance (TPR), defined as mPAP/CO, is used to distinguish elevated mPAP as an augmentation of CO in response to exercise versus pulmonary vascular remodeling or left heart failure [10]. Using a TPR cutoff > 3 WU at maximal exercise in combination with mPAP ≥ 30 mm Hg has both high sensitivity and specificity for the detection of a precapillary component of the PH, as opposed to Ipc-PH patients with a degree of Cpc-PH, may also exhibit an abnormal RA: PCWP that is >0.5 due to the involvement of right heart dysfunction in the setting of increased RV afterload.

## 3. CPET in the Assessment of PH

### 3.1. Precapillary PH

#### 3.1.1. Pulmonary Arterial Hypertension

Pulmonary arterial remodeling and vascular obstruction lead to increases in PVR and thereby mPAP. This gives rise to three main physiologic derangements: (1) ventilation/perfusion mismatch; (2) gas exchange abnormalities; and (3) increased RV afterload and reduced RV stroke volume [11]. These deficiencies explain the characteristic findings described during a CPET in a patient with PAH [Table 1].

In PAH, the ratio of minute ventilation (VE) to the production of carbon dioxide (V__co_2___) or Ve/V_co_2__ is demonstrative of inefficient ventilation during exercise. As such, patients with PAH experience a steeper Ve/V_co_2__ slope of this relationship. Under normal physiologic circumstances, there is some degree of V/Q mismatch at baseline, with the apices of the lungs receiving relatively less perfusion due to blood flow pumping against gravity. With exercise, the augmentation in stroke volume can more easily pump blood to the apices and there is an overall decline in V/Q mismatch. In patients with PAH, however, fixed obstructions in the pulmonary vascular bed, lead to attenuation in perfusion to ventilated alveoli that result in ventilation–perfusion mismatching and increased dead space ventilation. This is further reflected by an abnormal reduction in end tidal_co_2__ (Pet_co_2__). PAH patients have lower Pet_co_2__ from increased ventilation, reflected by increased VD/VT. Low Pet_co_2__ corresponds to ventilatory insufficiency and is proportional to the severity of peak VO_2_ (aerobic capacity) impairment. Low Pet_co_2__ is also inversely related to a rise in mPAP during exercise [12]. Pet_co_2__ levels < 30 mm Hg at an anaerobic threshold (AT) during exercise have been correlated with PAH [13,14]. Together, Pet_co_2__ and Ve/V_co_2__ can provide insight into disease severity based on the degree of ventilation–perfusion mismatch that reflects the underlying pathologic remodeling of the pulmonary vasculature.

Therefore, in patients with suspected early PH due to pulmonary vascular disease, CPET can be a highly useful screening tool. Patients with a Ve/V_co_2__ > 40 mm Hg and Pet_co_2__ < 30 mm Hg have been shown to be highly predictive of the presence of elevated PVR with exercise [15]. In a study by Raza et al., the subjects with both of these abnormal gas exchange parameters had an average PVR of 7.2. In those with one abnormal parameter, the average PVR was 4.2, and in those with both normal parameters, the average PVR was 2.5 [15]. Therefore, these gas exchange parameters can be used to guide providers on which patient needs further evaluation with RHC, either at rest or with exercise.

Arterial O_2_ desaturation during exercise is related to a widened alveolar–arterial gradient. This is caused by higher pulmonary blood through a decreased vascular bed, which reduces both red blood cell transit time and hemoglobin saturation. PAH patients have classically been described to experience oxygen desaturation of >3% from rest to peak exercise without a rise in Pa_co_2__ [12]. Desaturation is also possible due to the opening of an exercise-induced right-to-left shunt, which corresponds to very low Pet_co_2__ values at rest. Exercise-induced right-to-left shunt from a patent foramen ovale opening should be considered when there is a sudden decrease in Pet_co_2__ and oxygen saturation, and a sudden increase in end-tidal oxygen tension (PetO_2_), Ve/V_co_2__, and respiratory exchange ratio [13].

Many PAH patients have mechanical ventilatory abnormalities that contribute to dyspnea during exercise. Studies have demonstrated that a significant proportion of patients with PAH have (a) mechanical ventilatory deficits in tidal volume expansion and (b) decreases in inspiratory capacity and inspiratory reserve volume indicative of dynamic hyperinflation [14,16]. Patients who demonstrate dynamic hyperinflation during exercise will have more dyspnea at a given work rate than patients without hyperinflation [17].

As discussed previously, cardiac adaptation during exercise is reduced in PAH patients. Impaired CO at the initiation of exercise correlates with a transient decrease in stroke volume and is reflected on CPET by low peak O_2_ pulse (VO_2_/HR).

#### 3.1.2. Thromboembolic Disease

Abnormal exercise capacity of cardiopulmonary origin is common after a pulmonary embolism (PE). Recently, there has been growing interest in the use of CPET in patients who experience persistent dyspnea following PE treatment. Farmakis et al. recently demonstrated the prevalence of cardiopulmonary limitations that were unmasked with exercise in patients 3 months and 12 months following a PE [18]. In this prospective study of patients with acute PE, approximately 90% had evidence of ventilatory inefficiency and 30% had evidence of limited cardiocirculatory reserve. Importantly, the presence of ventilatory inefficiency following PE may speak to the development of chronic thromboembolic disease (CTED) and shed light on the potential role of follow-up imaging after PE and prognostication [19]. This has significant clinical implications, for this syndrome can often be treated with balloon pulmonary angioplasty (BPA) with improvements in CPET parameters including ventilatory insufficiency following this therapeutic intervention [20]. Therefore, CPET may serve as an advantageous diagnostic modality for patients with persisting symptoms after PE.

Another use of exercise testing is in CTEPH patients who have had a pulmonary thromboendarterectomy (PTE) and may have persistent PH following the surgery. Despite profound hemodynamic and clinical improvements in most patients post PTE, there is often residual PH that persists. This can be due to very distal thromboembolic disease in the pulmonary vasculature that is unable to be surgically removed or from a small-vessel pulmonary arteriopathy. It is thought that up to 45% of post-PTE patients have residual PH after PTE [21]. A subset of these patients will have mild resting PH and only mild RV dysfunction which may or may not be clinically significant. Here, the use of CPET can be highly valuable, for it can provide greater insight into the significance of each individual patient’s physiologic response to exercise. Exercise testing can therefore be utilized to differentiate those post-PTE patients who will derive benefit from pulmonary vasodilator therapy or BPA, depending on the reason for the residual PH [22].

### 3.2. Isolated PH and Combined Precapillary and Postcapillary PH

As above, increased left atrial pressure, whether secondary to systolic dysfunction, diastolic dysfunction or severe left-sided valvular disease, can result in elevated mPAP. It is important to note that noninvasive CPET cannot unmask an elevated left atrial pressure with exercise, for there is no direct measurement of left-sided filling pressures during this testing modality. Furthermore, unless extreme, a rise in left-sided filling pressure will not typically result in low breathing reserve or oxygen desaturation. A normal exercise capacity on CPET makes the presence of PH due to elevated LAP highly unlikely and can be useful to rule out this entity [23]. However, if there is reduced peak VO_2_ with evidence of cardiopulmonary limitation, this is not at all specific to elevated left-sided filling pressures. Therefore, RHC with exercise is preferred if suspecting EIPH due to left heart dysfunction.

Despite the lack of specificity in the diagnosis of Ipc-PH and Cpc-PH with CPET, there are certain trends unmasked with gas exchange testing, that can assist with differentiating between these two groups. Studies have found that peak VO_2_ and oxygen saturation have been shown to be lower in patients with Cpc-PH than Ipc-PH. Additionally, at the anaerobic threshold, Ve/V_co_2__ tends to be lower in patients with Ipc-PH than Cpc-PH, but this difference is diminished at peak exercise [24]. Perhaps the most useful finding to help differentiate between CpcPH and IpcPH is that of exercise oscillatory breathing (EOB). EOB has been reported many times to be absent in pure PAH but is a frequent finding in patients with a component of postcapillary PH. This speaks to the concept that it is likely increased PCWP that plays a role in the development of oscillatory respiration. In a study by Caravita et al., the prevalence of EOB increased from PAH (absence of EOB) to CpcPH (17%) to IpcPH (40%). Therefore, these patterns can help clue the provider in on the potential mechanisms of exercise intolerance in a patient presenting with dyspnea.

### 3.3. Invasive CPET

**Figure 1:**
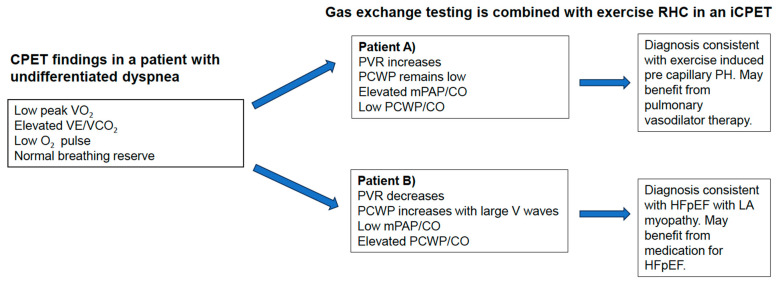
A diagram illustrating how invasive hemodynamics add insight into the concomitant findings on gas exchange testing. Here, the added hemodynamics unmask very different diagnoses despite having the same findings on CPET alone.

In those with mixed pre and postcapillary PH, both increases in LAP and PVR will contribute to increases in mPAP. Invasive RHC with concomitant gas-exchange testing can allow providers to identify specific patient phenotypes and differentiate those with predominant left heart disease versus primary PAH. There is certainly a convenience in performing hemodynamic and gas exchange assessment simultaneously. Hemodynamic evaluation, in addition to that of gas exchange patterns, can alert providers to cardiac subtypes of the disease process. Concomitant measurements of ventilatory inefficiency, anaerobic threshold, and several other parameters along with invasive hemodynamics, provide an additional layer of assessment and diagnosis of multifactorial dyspnea [25]. For example, Ve/V_co_2__ may be elevated in both precapillary PH and postcapillary PH. If hemodynamics are performed alongside gas exchange testing, however, the PVR and PCWP measurements with exercise can more closely tease out if there is a precapillary component to the PH (Figure 1).

In a recent study by Caravita et al., the authors analyzed the results of 86 HFpEF patients who underwent an invasive CPET in order to better understand the HFpEF pathophysiology and phenotype of exercise-induced pulmonary vascular disease in this cohort [26]. The findings of HFpEF-latentPVD phenotype (defined as a PVR > 1.74 with exercise) included lower peak VO_2_, lower stroke volume and CO augmentation, and lack of decrease in PVR as compared to their HfpEF control counterparts. There was also significantly higher systolic right atrial pressure in the HfpEF-latentPVD, which likely speaks to worsening TR with exercise. Understanding these important differences may have future clinical implications. There are currently no approved PH medications for Cpc-PH or PH in HFpEF. However, this may be due to a lack of appropriately categorizing these patients. By understanding each individual patient’s unique physiology, we may find that treatment can increase peak VO_2_ and CO, and reduce the degree of TR with exercise.

## 4. Exercise Stress Echocardiography

Noninvasive imaging exercise testing has been a long-established tool in the assessment of coronary artery disease and the evaluation of left-sided heart disease. There are now more studies exploring the role of exercise echocardiography in unmasking and phenotyping PH and right-sided heart pathologies. In a recent study by Gargani et al., subjects underwent transthoracic echocardiography (TTE) at rest and during exercise. Key measurements obtained at rest and exercise included tricuspid annular planar systolic excursion (TAPSE), systolic pulmonary artery pressure (sPAP), CO, PVR, and mPAP/CO. The study included elite athletes, patients with PAH (confirmed with RHC), patients with connective tissue disease without overt PH, patients with left heart disease, and patients with chronic lung disease [27].

Patients with left heart disease demonstrated the lowest CO during exercise, whereas patients with PAH had the steepest mPAP/CO slope. The lowest TAPSE/sPAP ratio was found in patients with PAH [27]. These findings suggest that there could be a potential role of exercise TTE in revealing early or EIPH based on these measurements. These parameters, combined with the more established measurements used in the assessment of diastology and left heart dysfunction, such as left atrial volume index and E/e′, can be used to phenotype patients into precapillary, postcapillary, and Cpc-PH categories.

## 5. Prognostication

There are several CPET findings that have been shown to predict prognosis in several types of PH. Wensel et al. found that peak exercise systolic blood pressure of less than or equal to 120 mm Hg and peak VO_2_ less than 10 mL/kg/min were associated with worse survival [28]. Ventilatory inefficiency, often represented by the Ve/V_co_2__ slope, has been repeatedly demonstrated to be associated with survival in PAH and CTEPH. For example, in a study including both patients with PAH and patients with CTEPH, a slope greater than or equal to 60 revealed a very high risk of death at two years [17]. The association of increased Ve/V_co_2__ with cardiovascular risk also holds true in patients with chronic heart failure both with systolic and diastolic dysfunction [29].

There have also been invasive CPET variables that are important prognosticators, particularly in patients with left heart disease. As mentioned in an above study, patients with HFpEF who demonstrated an elevated PVR with exercise, labeled as HFpEF-latentPVD, had a reduced event-free survival as compared to their HFpEF counterparts [25]. Additionally, studies have illustrated the prognostic value of distensibility, for patients with lower distensibility have worse overall survival [5]. These findings provide insight into the progression and stages of each patient’s disease process. In the realm of exercise stress echocardiography testing, Gargani’s study also found a significant decrease in mortality-free survival in patients with a mPAP/CO slope > 5 Hg·min/L as well as in patients with a TAPSE/mPAP < 0.7 mm/mm Hg [26].

Taking these findings together, there is a clear and important role of exercise testing in patients with PH given the many prognostic implications.

## 6. Future Directions

Future studies should further investigate the role of CPET in PH patients with precapillary disease who have multiple risk factors, including left heart disease and chronic lung disease. Determining when to challenge PAH-specific therapy in select patients with multiple age-related comorbidities remains an art that is difficult for many PH specialists. Specifically, determining (1) the reliability of CPET in differentiating the impact of multiple risk factors on the RV and pulmonary circulation and (2) the response to specific interventions in PH patients with multiple age-related co-morbidities. Another study direction should investigate the reliability of noninvasive imaging versus invasive hemodynamic testing during CPET testing.

## 7. Conclusions

Cardiopulmonary exercise testing is of particular use to identify mechanisms of exercise intolerance or chronic dyspnea when the etiology is unclear. It is paramount to understand the pathophysiological abnormalities, during CPET, to recognize this modality’s wide breadth of utility in PH patients. Its value is additive when combined with invasive hemodynamic monitoring or noninvasive imaging during exercise testing. There is a growing role for its use in PH patients of any classification. CPET can be used to suggest the presence of pulmonary hypertension; elucidate drivers of dyspnea in patients with secondary pulmonary hypertension (i.e., from underlying chronic lung disease, left heart disease, chronic thromboembolic disease); and provide prognostication in PH patients to direct goals of care discussion and determine the role of definitive therapy.

## Figures and Tables

**Table 1:** Commonly found CPET findings in pulmonary arterial hypertension patients.

Reduced Variables	Increased Variables
Peak VO_2_ (<20 mL/kg/min)	Ve/V_co_2__ slope (>30)
Work Rate	A-a O_2_ differences during exercise (can be over 45 mm Hg)
VO_2_/Work Rate (<10 mL/min/W)	VD/VT during exercise (>30%)
O_2_ pulse	
O_2_ desaturation during exercise (>3% without Pa_co_2__ rise)	
PET_co_2__ at rest and anaerobic threshold	
Anaerobic threshold	

Ve: minute ventilation; VO_2_: oxygen uptake; VD: dead space volume; VT: tidal volume; A-a O_2_: alveolar–arterial oxygen tension difference; PET_co_2__: end-tidal carbon dioxide tension.

## Data Availability

Not applicable.
